# Reconsidering the dangerous normalization of postpartum sleep loss

**DOI:** 10.1007/s00737-026-01698-y

**Published:** 2026-04-14

**Authors:** Alexandra M. Davis, Natalie L. Solomon

**Affiliations:** 1 PGSP-Stanford Psy.D. Consortium, Palo Alto University, Palo Alto, USA; 2 Department of Psychiatry and Behavioral Sciences, Stanford University School of Medicine, Stanford, USA

To the Editor

Almost all new parents experience sleep disruption in the postpartum period. Feeding, caring for, and soothing an infant throughout the night are necessary aspects of early caregiving and contribute to the near-universal experience of postpartum sleep disruption. The mismatch between adult sleep-wake patterns and those of an infant often reduces both the quantity and quality of sleep for new parents. Opportunities for adults to extend their sleep window beyond nighttime hours are often limited. This is particularly true in individualistic societies like the United States, where one or two parents typically bear the full burden of infant care.

It is well established that sleep loss negatively affects mental and physical health, and postpartum sleep loss is associated with an increased risk of depression, mania, psychosis, suicidality, and child abuse (Lewis et al. 2018; McQuillan et al. 2019; Okun and Lac 2023). A smaller subset of this population may develop postpartum insomnia. Insomnia is characterized by difficulty initiating sleep, maintaining sleep, or returning to sleep, despite adequate opportunity for sleep. This disturbance must occur at least three times per week, for a minimum of three months, and must be accompanied by functional impairment and/or clinically significant distress. In the postpartum period, sleep difficulty that continues after caregiving demands have subsided may indicate an insomnia disorder rather than sleep disruption driven by infant care. While all forms of sleep loss can adversely affect mental health, insomnia carries distinct risks, including stronger associations with psychiatric disorders, physical morbidity, and increased risk for chronic insomnia (Riemann et al. 2017). The differences in risk may partly be explained by the hyperarousal processes that uniquely characterize insomnia (Riemann et al. 2017).

Unfortunately, distinctions between postpartum sleep loss related to caregiving and postpartum insomnia are not consistently recognized in clinical practice or research, in part because of nonspecific assessment tools and gaps in conceptual understanding. Common instruments such as the Insomnia Severity Index (ISI) and the Pittsburgh Sleep Quality Index (PSQI) often fail to capture whether an individual has adequate sleep opportunity. As a result, postpartum sleep loss due to caretaking is often conflated with postpartum insomnia. Quin and colleagues conducted a study examining the relationship between DSM-5 insomnia criteria and peripartum populations, which found that a failure to consider sleep opportunity can result in a two- to fourfold overdiagnosis of Insomnia Disorder during the perinatal period (Quin et al. 2022). Paradoxically, this can result in a minimization of insomnia disorder, making it seem as if this is a ubiquitous and thus normal part of the postpartum period.

Collectively, these findings underscore the need for more nuanced, postpartum-specific tools that can accurately distinguish insomnia from sleep disruption due to caretaking. A central assessment question in identifying postpartum insomnia is: “When your baby does not need your care, are you able to sleep?” A ‘yes’ response may suggest that sleep loss is attributable to external demands such as infant feeding, pumping, changing, or soothing. A ‘no’ response may indicate insomnia symptoms. OBGYNs can use this brief screening question to differentiate types of sleep loss and inform appropriate intervention strategies (Fig. 1).

**Fig. 1:**
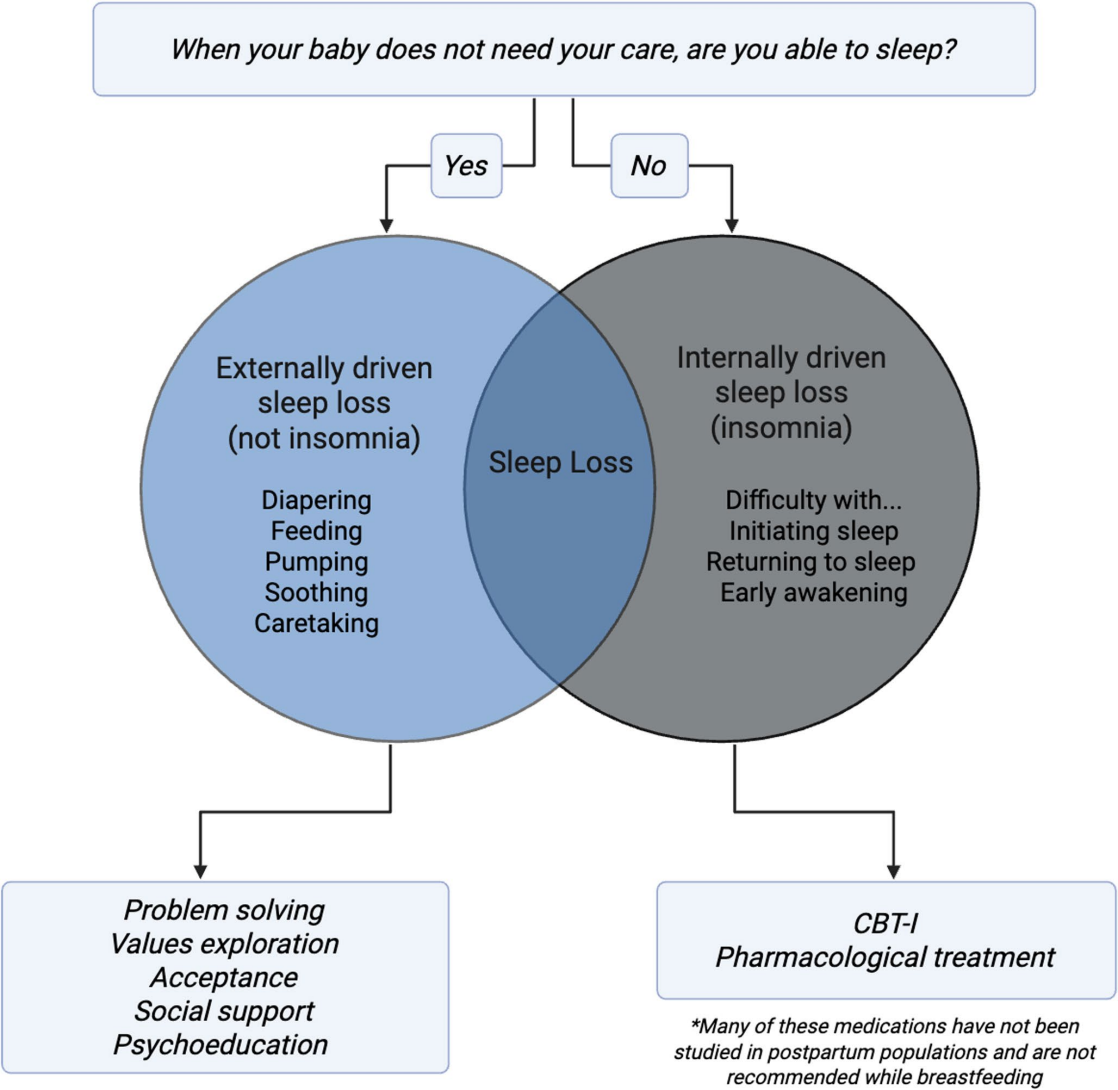
A Clinical Guide for Addressing Postpartum Sleep Loss

Sleep loss is not monolithic, and thus, requires a variety of interventions. Psychoeducation about infant sleep and development, alongside traditional CBT-I psychoeducation on sleep architecture, hygiene, and sleep processes, can help normalize early caregiving demands and reduce distress for new parents. Acceptance and values-based approaches may also support parents in identifying what they can control, where to direct their energy, and when to seek help. Social support and problem-solving strategies can redistribute caregiving responsibilities, such as alternating nighttime duties or dividing tasks like feeding and diapering, to create more opportunities for rest. Cognitive Behavioral Therapy for Insomnia (CBT-I) is the gold standard treatment for chronic insomnia and targets the behaviors and cognitions that sustain sleep disturbance. Evidence suggests CBT-I during pregnancy can reduce insomnia symptoms and lower risk for postpartum insomnia, though few studies have examined its use during the postpartum period (Manber et al. 2019). Most pharmacologic treatments have not been well studied in postpartum populations and may be less desirable for individuals who are breastfeeding.

Almost all postpartum individuals experience sleep disturbances, which can negatively affect both parental and infant health. A subset will develop clinical insomnia, carrying heightened risks for parent and child. More research is needed to clarify the prevalence of postpartum insomnia, understand its distinct features, develop context-specific assessment tools, and identify effective interventions.

## Data Availability

No datasets were generated or analysed during the current study.
